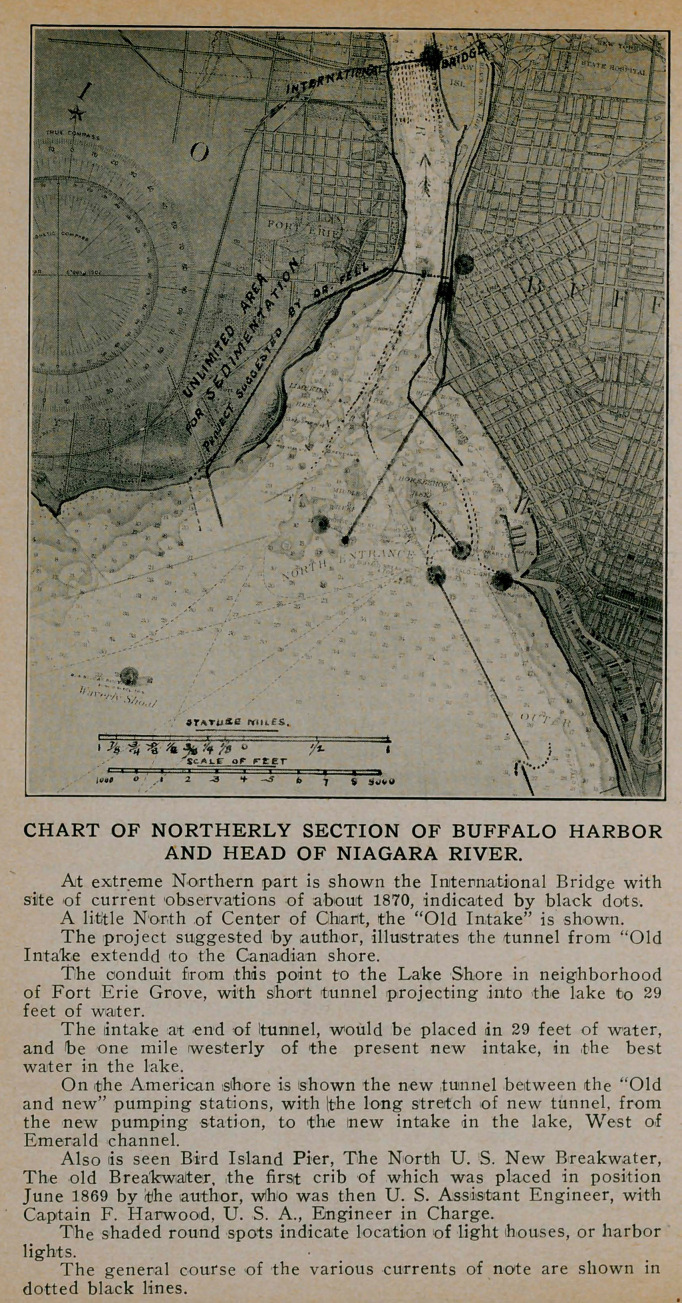# Sanitation of the Cities of the Niagara Frontier

**Published:** 1912-04

**Authors:** George Edward Fell

**Affiliations:** Buffalo, N Y.; Formly U. S. Assistant Engineer,* Construction of Harbor Works, Lake Erie. Assistant Engineer, City of Buffalo, N. Y. Following graduation in medicine, President American Microscopical Society, Professor of Physiology and Microscopy Medical Department of Niagara University, Physician Sisters of Charity Hospital, Assistant Surgeon, Charity, Eye, Ear and Throat Hospital, Etc.


### Sanitation of the Cities of the Niagara Frontier. The Buffalo Waterworks Controversy.

GEORGE EDWARD FELL, M. D., F. R. M. S.
Buffalo, N Y.

Formly U. S. Assistant Engineer,* Construction of Harbor Works, Lake Erie. Assistant Engineer, City of Buffalo, N. Y. Following graduation in medicine, President American Microscopical Society, Professor of Physiology and Microscopy Medical Department of Niagara University, Physician Sisters of Charity Hospital , Assistant Surgeon, Charity, Eye, Ear and Throat Hospital, Etc.

THE editorial of this JOURNAL for January 1912, refers to the possible contamination of our intakes by refuse thrown from vessels, and also from the hulls of the vessels, passing in and out of Buffalo Harbor. It suggests a scheme impractical and remarkable in the preparation of new channels, or the movement of the intakes to peculiar locations.

To prevent "roiliness" there is suggested the construction of another breakwater, at right angles to present one, to provide "quiet pools" which could be dredged repeatedly from the accumulated material which might fill them. A breakwater in this position would not afford a "lee" or quiet the water in a S.W. gale. We cannot reconcile the idea of the silt settling to the lake bottom, when every wave produces a disturbance to a greater or less degree of every particle of water to the greatest depth of this shoal lake. As long as the winds blow, we cannot prevent the suspension in our lake water of the accumulated bottom debris known as silt.

### CAUSE OF THE "ROILINESS."

Silt the result of animal, vegetable and mineral substances which have gained acces sto the water of the lake and been reduced to an impalpable powder, is found everywhere on the bottom of the lake where no marked current exists. It is held in suspension in the water in gales and we would be no better off so far as it is concerned, if we had an intake constructed, half way between Sturgeon Point and Point Abino, or in the middle of the lake.

On this account for the purity of our water from this non-pathogenic debris,** we will absolutely require a sedimentation basin, or require that every individual filter the water drawn from each tap in stormy weather.

Sedimentation will give us non contaminated pure water, if taken from the sites of the present intakes, but assuredly not

---

* civil.

** All evidences of value indicate that the water of Lake Erie, outside of littoral contamination is pure and free from pathogenic factors, when naturally sedimented by calm weather.

pure, if seepage of the impure Buffalo River water contaminates it before it reaches the wells at the shore ends of the tunnels.

### MEDICAL KNOWLEDGE NOT ENGINEERING KNOWLEDGE.

Allow me right here, to express an opinion regarding this controversy, which is, that medical men without special engineering experience, are not qualified to pass judgment upon engineering problems. Furthermore appended is a statement of the work with which the author was associated as hydrographical engineer, in the earlier days of his life, all of a most practical character, to indicate whether it should be assumed that he was so qualified.

In June 1869, the writer as U. S. Asst. Engineer, had direct charge of the placing of the first Crib of the great Breakwater system of Buffalo Harbor, having located the buoys for its location by triangulation. For two years previous he had been employed in connection with the extension of the old light house, South U. S. pier. He was employed in the actual observations of the currents of the Niagara River for the purpose of determining the location of the draw of the International Bridge, which demonstrated positively that the currents follow *closely* the axis of the river a matter of great importance, in this controversy. He made extensive surveys at Buffalo, Dunkirk, Erie and other harbors. In 1873-4, he made the survey for the "Harbor of Refuge" at Cleveland, Ohio, making the observations of the lake bottom at Cleveland, as well as Buffalo Harbor with a steamboat of his own devising, which admirably answered the purpose.

Subsequent to graduation in medicine, he has been a close observer of the changes taking place in and about Buffalo Harbor, being associated with the Crystal Beach Steamboat line for a number of years.

### EXPLANATION OF THE AUTHOR'S INTEREST IN THE CONTROVERSY.

In 1903 he made the *first* extended study and survey of the currents at the Easterly end of Lake Erie and head of the Niagara River. This was reported in great detail to Mayor Erastus C. Knight. His views were strongly opposed by the health authorities of the State and City, and the great majority of physicians and citizens, who, led by Engineer Fuller of New York City, differed from the author as to the danger of infection of the site of the old intake, at Massachusetts Ave., from the typhoid epidemic at Smokes Creek, and the sewage from Buffalo River. This was the principal question upon which hinged the need of new extensive water works for the city.

The author contended that the physical conditions, principally the currents at this part of the lake, influenced in their course by the outflow over Niagara Falls, as demonstrated by his extensive survey, prevented infection of the site of the intake.

Engineer Fuller and those associated with him succeeded in putting through the "seven million" dollar, new water works system. Subsequent investigation by celebrated experts, employed by the Buffalo Chamber of Commerce, have demonstrated that in many ways the New Water Works system was a great mistake, agreeing or showing that the contentions of Dr. Fell in 1903 were fully justified.

SOME QUESTIONS REGARDING THE CURRENT OBSERVATIONS.

My first demonstrations made in March and April 1903, in an Easterly gale, resulted in showing that the current starting in the course of the U. S. range lights for vessels entering the Niagara River between the "Dummy Light" and the Canadian shore, would pass close to, but Westerly of the old intake. No gales, Easterly or Westerly, can greatly modify the course of this current, *the proximity of the Canadian shore preventing it.*

The great mass of water entering the river follows the course of this deep channel current, whereas the entrance of the river and lake currents Easterly of the Dummy light, is comparatively shoal, clear to the American shore. This demonstrates that the current West of the Dummy, light, dominates the general entrance currents from the lake, and prevents with the increased outflow and necessarily increased current in the river in high water, *Southwesterly gales,* up lake currents at the Northerly and Southerly ends of the New Breakwater such as Dr. Allen J. McLaughlin* suggests as probable in extra heavy Southwesterly gales, with oscillatory fluctuation of the water of the lake. The entire length of the New Breakwater comes within the influence current of the Niagara, the controlling factor here. Please bear this in mind, as these important factors in the control of the currents were not sufficiently amplified in my report made to the City of Buffalo in 1893, or the review published in the Journal of the American Medical Association, September, 1910.

THE GREAT TEST CURRENT OF THE CONTROVERSY.

The current, Southerly and Easterly around the South end of the "New North Breakwater" has quickly carried me in a small boat, on the West side of breakwater Southerly, along the southerly one-third of the breakwater to the south end around into the harbor. Under any condition of gales, it is believed this current is constant as to direction. The writer has photographs which indicate its course when outlined with floating ice. From hundreds of personal observations I do not believe it has changed since the first crib of this breakwater was put in place. The physical conditions existing if carefully studied, simply explain the

* See Bulletin No. 67, page 42, Marine Hospital Service of the United States, by Dr. Allen J. McLaughlin.

cause and course of these currents as demonstrated by the deep water float observations.

The influence of a gale of wind exerted on the crests of innumerable waves, which produces moving water to the greatest depths in a shoal lake, drives it as a body in the direction of the wind, until the rise is sufficient to produce an oscillation, which lowers it. That such a fall takes place with sufficient rapidity to entirely overcome the influence of the increased current, under such conditions, of the Niagara River at the North U. S. Breakwater, from my observations, I cannot believe.

Now if these statements be true, they can be verified any time the currents suggested by Dr. McLaughlin, do not take place, and our new intake, as well as the old, is in no danger of contamination from shore currents carrying the foul typhoid debris of Buffalo River water. The opinion of those who have been active workers in and around Buffalo harbor for years, not interested in the promulgation of the new water works system, agree with me in these views.

### WHAT NATURAL CONDITIONS COULD HAVE GIVEN US.

The following plan (See Chart) indicates how all of these questions viz: possibility of infectious contamination of intakes from either the American or Canadian shore, infection through seepage of foul water over the tunnel conduits, and the question of adequate acreage for sedimentation purposes, ideal in character as to site, location and expense could have been eliminated from this controversy.

It is put forth with the purpose of strong criticism of the factors which should be permitted to rush through a scheme of improvement so vital to the interests, not only of a great city, but an additional large populous territory, when there was so much of apparent merit in the reasons for the disinterested opposition to it by the author and those who honestly supported his views.

This plan is well within the practical possibilities, it would be of international value, would have provided an ample site for the absolutely needed sedimentary basin, or even filtering plant, if needed. It would give us a much better and the most satisfactory site for the intake, which could be connected with much less length of tunnel, more ideally located, and at a saving of millions of dollars. It was right before the expert savants, who conceived and constructed the present new system, which will ever prove troublsome and the great disaster of last Summer may prove that it was not needed for years to come.

### WHAT MAY YET COME OR MIGHT HAVE BEEN.

It is this—extend the old waterworks tunnel, from old intake to the Canadian side of the river, and make it impervious to seepage a simple matter; raise it to an open covered conduit to be carried westward to the table land, now farm land, where there is no interference with the extent of the size of the sedimentary basin, pumping works or whatever adjuncts may be needed in the practical working of the plan. Utilize the present pumping facilities at Massachusetts Avenue, with practically no great change for the pumping of the pure sedimented water, from the new up lake intake.

This could be placed a mile further up the lake than the present new "Emerald" channel intake, and would have no connecting tunnel with a possible seepage distance of foul pathogenic, Buffalo river water of about three-quarters of a mile passing over it as is the condition with the new elaborate and already partially constructed scheme with its two miles of expensive tunnel construction already built.

### MINIMUM LENGTH OF TUNNEL REQUIRED.

There would be required by this plan but three-fifths of a mile of tunnel, including that in the Niagara and to connect with the intake in the lake. This intake would be in 29 or 30 feet of water instead of in 19 feet. It could be a mile further up the lake than the present new intake well away from any Canadian or American shore contamination. There would be seven-eighths of a mile of covered shore conduit, with 3,500 feet of sedimentary basin of any width desired. This would be located on practically level ground and could have been purchased or obtained from the Canadian Government as it could be made mutually as well as internationally satisfactory.

### WHAT IT WOULD ACCOMPLISH. IS IT PRACTICAL AND FEASIBLE?

Its simplicity indicates that there is nothing impractical or difficult in the project as suggested. Millions of dollars could have been saved the city of Buffalo, had the great rush and indefensible hurry to spend the money of Buffalo's citizens, have been halted in 1903.

We have nothing in our partially completed plan which in any way compares with that I have suggested in its absolutely answering the necessities of the case. It is the most feasible method of settling the question of the supply of pure potable water to the citizens of both nationalities, at the Easterly end of Lake Erie, and Niagara frontier, for all time to come.

We do not know what the future may bring forth, but the question arises, whether it would not be better to construct this new project, build the five-eighths of a mile of tunnels, with a temporary conduit connecting the two, which later could be connected with the sedimentary basin, than it would be to spend more millions in patching up the present project with no accessible territory for a complete sedimentation obtainable.

### THE MILITARY ASPECT WOULD NOT BE CHANGED BY THE NEW PROJECT.

The military aspect and international features are not at all new along the lakes; how many U. S. channels have been constructed in Canadian waters? The "Soo" locks at St. Mary's River are utilized by both countries; electricity manufactured in Ontario, is marketed in New York State, and is a no more marketable commodity than water.

What is most interesting, I am credibly informed *that our New Intake is in Canadian Water,* so that we are really taking water from foreign territory as it is.

The value of the desirable combination, the City of Buffalo with its unlimited money, as we understand and, the ideal farm land of the Fort Erie territory, and splendid location for intake would result most satisfactorily for the citizens of both countries.

This much for a might have been, possibly future project infinitely better than the one hastily and unwisely adopted by the City of Buffalo.

THE AUTHOR'S CURRENT OBSERVATIONS.

When my current observations were made in 1903, the best motives only influenced me. The results demonstrated, the currents working Easterly between the breakwaters, and in the same direction south of the new North Breakwater, was an interesting revelation.

Without specially considering its importance, I had noticed the latter from the time of the location of the very first crib of the North Breakwater and during its construction.

I was astounded by the assumption on the part of Engineer Fuller that I was wrong, because I could not believe that any other conclusion could be arrived at as the physical condition plainly points to the cause of these currents. The matter is not at all complex when the subject is honestly investigated.

But when I read his report and the manifest futile efforts he made to bolster up an argument to prove clinically that the Buffalo water *intake location* supply was contaminated from Smokes Creek and Buffalo River, and not through seepage, and other well-known sources, and found that no one seemed to agree with me. When the fatal project for the welfare of Buffalo was adopted and pushed to completion I took it cooly, for I believed a day of recokening would surely come as it has, and that the truth would some day prevail.

THE AUTHOR'S VIEWS IGNORED.

To show that my opinion was of no account, when my bill was presented to the city, recommended by Mayor Knight, who saw the force of my arguments, and believed then as now that they were right, it went to the committee of the aldermen and was discussed.

Ex-Mayor Adam, then Councilman, took special delight in opposing it, and asked what value my work was to the City of Buffalo. I explained that it indicated that our intake was not contaminated by Smokes Creek or Buffalo River water; that it was not necessary to construct a great new water works system, etc., but it had no effect. My bill of $600, was cut to $200, not enough to meet my expenses; my attorney informed me that even with the Mayor approving it, it could not be collected, so that I necessarily accepted it. The city paid for this service at the rate of about $2.00 per day when it at the same time was paying Mr. Fuller $75.00 per day. It would have paid the City of Buffalo to have paid Mr. Fuller one million dollars and J. N. Adam another million, and held up the report of the board of engineers which made the plans for the extensive tunnel scheme than to have gone through with it, with the existing results. With tunnels that permit of seepage of Buffalo River water, so that we can never be positive that our supply is not contaminated. A tunnel that will fill as rapidly as the nine foot tunnel did, and might not stand up against a forty-five foot pressure is not safe to depend upon for a pure water supply, when unquestioned typhoid contaminated water is running over it.

### THE CAUSE OF THE CONSTANT CURRENTS AT THE HEAD OF THE NIAGARA.

There is no uncertainty here, the enormous outflow of the Niagara River is the factor which influences and produces the peculiar courses of the currents at the Buffalo end of Lake Erie.

This great factor has been studiously ignored and placed in the background by the advocates of the great unnecessary additional water works system at Buffalo, from Engineer Fuller, his partner Rudolph Herring, to the Committee of the Erie Couny Medical Society which echoes the opinion of Dr. Allen J. McLaughlin of the Marine Hospital service expressed in the presentation of a chart in his "Bulletin No. 77." From my many years of personal experience sailing in and around Buffalo Harbor, I cannot reconcile his views with the other features of his most valuable report.

### THE OUTFLOW OF NIAGARA RIVER.

From the report of the engineers who constructed the International Bridge at Buffalo, N. Y., along about the year 1870, and from a series of current observations which as (U. S. Assistant Engineer) and transit man, I assisted in making, it was determined that under ordinary conditions there are 245,000 cu. ft. of water per second passing down the river. Assuming this to be the case we have 1,575,000 galls. per second—94,500,000 galls. per minute—5,670,000,000, per hour or the enormous quantity of 158,347,612,800, over one hundred and fifty-eight billion gallons per day of twenty-four hours.

And yet as I listened to the testimony of Mr. Herring on the subject of the contamination of our old and new intakes, during the so-called "Fisher investigation" he failed to mention this great factor as of any importance regarding the question, when it is really the *paramount* one. It makes our condition so far as the influence of the currents goes different from that at any other point on the great lakes, and accounts for the immunity of the intakes from shore pollution.

To form an opinion of this great outflow, we may state that more water passes from the entrance of Lake Erie into the Niagara River in two minutes, than is pumped into the whole Buffalo city water supply in twenty-four hours.

When the lake rises in heavy gales the current in the Niagara River may run as swiftly as twelve miles per hour, according to Col. Gzowski, the engineer of the International Bridge, and outflow is enormously increased, thus tending the more to retain the general constant course of the currents, notwithstanding the fluctuations, or rather oscilliations of the waters of the lake by Western gales.

TABLE NO. XII, OF ENGINEER FULLER'S REPORT.

Another point according to Engineer Fuller's Report to the City of Buffalo, which shows some questionable and unexplainable findings is that over three thousand feet of the new tunnel, between the new broken down pumping station and the new intake at Emerald channel, has the foul typhoid water of Buffalo River passing over it.

What a great chance for seepage to make our potable water much more dangerous than ever before if this great tunnel is not water tight. Is this tunnel any better in its construction, than the nine foot one between the two pumping stations? is a question of vital importance.

Again this table No. XII is supposed to show the results of bacteriological tests of the Lake Erie water, four feet below the surface, on a still day in August. One mile out in the Lake at Stony Point it is very good or ("25 c.c."). It reports very good water also half way between the "Dummy light" and the North end of "old breakwater," also very good water half way between Bird Island Pier and the old intake, thus granting a factor of safety, so far as any danger of contamination from sewage by the old intake is concerned of about 350 feet, a pretty good allowance in a current running from seven to twelve miles per hour. But the remarkable feature is that from Smokes Creek, entirely to the North end of the Old Breakwater, the red light, the water is bad, bad, or "5% c.c." water. This feature is unexplainable, and from the physical conditions which causes the current to pass from the lake to the harbor along this entire breakwater, it cannot be true.

Was it intended to show that Smokes Creek in quiet August weather, when there was virtually no flow from it befouled the great body of water, west of the over three mile stretch of breakwater, notwithstanding the great body of water passing from the lake to inner harbor through the South Channel Entrance and coming from the admittedly pure (25 c.c.) water one mile out in the lake at this point. This channel entrance is about six hundred feet wide, by thirty feet in depth and is supposed to be brushed aside by the infinitesimal Smokes Creek outflow. Such bacteriological examinations are worse than useless:

### BACTERIOLOGICAL TESTS NEEDED.

Honest impartial bacteriological tests of similtaneously collected samples of water, from the intake end, and from the wells at the shore ends of the tunnels, where seepage may have affected the water in its passage through the tunnel, should be systematically made and kept up for a considerable period of time Summer, Winter and also Spring, when the freshets are throwing the over filled streams and sewers with contamination of all kinds into the river and lake.

It is these natural conditions which will prove who has been right in his opinions. A grand and unanswerable demonstration of this character was made by nature in this present controversy in March 1903, when the whole typhoid debris of Smokes Creek and Buffalo River, full of sewage and typhoid "scourings," was washed out into the lake and Niagara River, when it was predicted by the advocates of the unsafe location of the old intake, that a terrible epidemic would prevail, and yet during this time and following it, the percentage of typhoid in the city *continually decreased.*

Nature will not deceive us, and yet, notwithstanding this unquestionable proof of the purity from contamination of the old intake site from Buffalo River and Smokes Creek, the advocates of the seven million dollar project still claimed that it was located in a dangerous location. I have been and am willing to abide by the verdict which the natural conditions at this end of Lake Erie will present as the years go by, and do not fear the result.

### THE MUDDY CLAY COLORED WATER OF LAKE ERIE.

Another fact which I have not seen mentioned in this enquiry of considerable importance macroscopically, pertains to the color of the Lake Erie water in the neighborhood of the Emerald Channel.

Some prominent persons have inferred that the clay colored water which has been seen at times to pass from the region of the Emerald channel towards the old intake was evidence that Buffalo river water contaminated the old intake. I have observed this several times, but as this part of the lake was for fifteen or twenty years, the dumping ground for thousands of tons of material, principally red clay, dredged from the slips and dock construction of the inner harbor, we can readily dispel the idea that such color is associated with Buffalo river water contamination. Under wind and ice disturbances, this soft clay is carried into suspension in the water, and serves often to make our drinking water more like that of the Mississippi than our ideal idea of Lake Erie water.

It has thus been the custom of those who advocated the new waterworks system to ignore either through ignorance or no desire to know the actual physical conditions which would aid in proving the safe character of the water at the old intake site.

They were not seeking, but evading the truth.

My current observations were ridiculed, but as time demonstrated the value of the truths unfolded by them, the army of investigators made good use of the report to Mayor Knight, adopted their findings, without giving the first demonstrator of this interesting natural condition the least credit for his work.

SOURCE OF TYPHOID FEVER AT BUFFALO.

By the pessimistic commission of the Erie County Medical Society, about all the typhoid fever is credited to the water supply, which twenty-five years ago would have been looked upon as reasonable.

No credit is given the *Musca Domestica,* the human typhoid carrier, or the nearness of one of the greatest typhoid centres in the country, Niagara Falls and adjoining cities, contaminated by the sewage of Buffalo, or any consideration given the large moving summer population along the Eastern shore of the river, directly north of Buffalo, which revels in the sewage always along this shore. Again there should be obtained so far as possible the source of contagion affecting the patients of the city.

Some years ago I took the trouble to enquire of three haphazard convalescent patients as to the source of their fever.

One had been in Pittsburg, another at Niagara Falls, and the third had been travelling between Tonawanda and Buffalo, these were undoubtedly statistically credited to our water supply.

SUM IT UP AFTER CAREFUL STUDY.

It amounts to this—if the tunnel from the new intake is free from seepage, the water pumped to our city supply will be as good as can be obtained at any point in easterly end of lake. If not, the construction of new tunnel and intake was useless or worse, as a *better,* feasible and *much less costly* plan could have been provided, without this danger, as I have shown, or just as good water could have been obtained at the shore end of the old intake tunnel. In this event a filtration plant on account of the seepage, would have been required, or only a sedimentation basin if the tunnels were made impervious to seepage.

FINIS.

To understand this subject clearly, it must be studied in detail. My report to the city, Common Council proceedings, June 29, 1903, paper in the *Journal American Medical Association,* September 3, 1910, are suggested.

To those who believe that Lake Erie is fast becoming a "Foul cess pool" I commend the "Report of Streams Examination," Sanitary District of Chicago, Chemic and bacteriologic examinations, made under the direction of Arthur R. Reynolds, Commissioner of Health Chicago, and give heed to its conclusions. The panacea for all is,

PREVENT THE BEFOULMENT OF ALL NATURAL POTABLE WATER WAYS.

See paper by the author, *Journal American Medical Association,* December 23, 1911. This good work is being systematically carried out by the disinfection of stools at the bedside, and the following paper is really the beginning on a systematic plan of the prevention of befoulment of potable water ways, and as I long ago suggested, sensibly begins at the bed-side of the patient—the first point in the battle against typhoid fever.* This, with the anti-typhoid vaccination, so successfully adopted by the U. S. Army and beautifully set forth by Major Frederick F. Russell, U. S. A., at the meeting of the Medical Union in this city last January, will result in the virtual banishment of typhoid fever from every civilized country on the globe.

## Figures and Tables

**Figure f1:**